# Bridging Three Gaps in Biodegradable Plastics: Misconceptions and Truths About Biodegradation

**DOI:** 10.3389/fchem.2021.671750

**Published:** 2021-05-14

**Authors:** Shinhyeong Choe, Yujin Kim, Yejin Won, Jaewook Myung

**Affiliations:** ^1^ Department of Civil and Environmental Engineering, KAIST, Daejeon, South Korea; ^2^ Department of Systems Biotechnology, Chung-Ang University, Seoul, South Korea

**Keywords:** bioplastics, biodegradable plastics, biodegradable products, misconception, sustainability, biopolymer, biodegradation

## Abstract

In the wake of plastic pollution increasing around the world, biodegradable plastics are one of the fastest-growing segments within the global plastics market. The biodegradation of these plastics depends on diverse factors including, but not limited to, the physicochemical structure of the materials, environmental conditions, and the microbial populations involved in the biodegradation. Although laboratory-based biodegradation tests simulate natural processes, they cannot precisely mimic the natural biodegradation of biodegradable plastics due to the disparity of several factors. In addition, the biodegradation levels claimed and/or reported by individuals and studies in different environments vary to a great extent. Biodegradable plastics are considered a sustainable alternative to non-biodegradable conventional plastics and are being promoted as an eco-friendlier choice for consumers. However, biodegradable plastics might not be as biodegradable as commonly believed, particularly in natural environments. This mini-review aims to bridge the following three gaps in biodegradable plastics by elucidating the common misconceptions and truths about biodegradation: i) the gaps among reported biodegradation level of biodegradable plastics; ii) the gaps between the biodegradation conditions in the controlled laboratory system and in the natural environment; and iii) the gaps between public perception and the actual environmental fate of biodegradable products. These gaps are critically reviewed with feasible solutions. This work will ease the assessment of biodegradable plastics and provide sound communication on corresponding claims–a prerequisite for successful market performance.

## Introduction

The massive accumulation of plastics in the natural environment is threatening the sustainability of our planet (Jambeck et al., 2015; UN Environment, 2018). As of 2014, over 250,000 tons of plastics were estimated to be floating in the sea (Eriksen et al., 2014). It is predicted that by 2030, 90 Mt/year of plastics will enter the aquatic ecosystem under the scenario where the current plastic production trend continues without improvements in the waste management system (Borrelle et al., 2020). Bioplastics refer to synthetic polymers that are biodegradable [e.g., poly(lactic acid) (PLA)] and/or are derived from bio-based materials [e.g., bio-based poly(ethylene) (bio-PE)]. Biodegradable plastics are one of the fastest-growing segments within the global plastics market. The global production capacity for biodegradable plastics was 1.2 Mt/year in 2020 and is expected to grow rapidly (European Bioplastics, 2020).

Numerous standards have paved the way for evaluating the biodegradability of plastic by simulating various environments, including natural conditions such as soil and aquatic environments, and systemically controlled conditions such as industrial composting and anaerobic digestion. Studies on the biodegradability of biodegradable plastics have been conducted according to the most prominent standards such as International Organization for Standardization (ISO), American Society for Testing and Material (ASTM), and European Norm (EN) (Eubeler et al., 2009). Although laboratory-based biodegradation tests simulate natural processes, they cannot precisely mimic the natural biodegradation of biodegradable plastics due to the disparity of several factors. In addition, the biodegradation levels claimed and/or reported by individuals and studies in different environments vary to a great extent.

Despite of legislative efforts for the standardization and specification of biodegradable plastics, the complicated descriptions and coverage of bioplastics and biodegradable plastics (e.g., bio-based, biodegradable, compostable, oxo-biodegradable plastic, etc.) are confusing to the public. The word “bioplastic” is commonly perceived as a synonym for “biodegradable plastic” (Dilkes-Hoffman et al., 2019). In addition, a common perception of biodegradable plastics is that the materials will biodegrade in natural environments. The reality is that many “so-called” biodegradable plastics are not biodegradable in the aqueous environments (Bagheri et al., 2017; Emadian et al., 2017).

Most countries label the products decomposed under controlled composting test within the designated period. Some examples of these test standards are ASTM D5338 in the United States, EN 13432 in European Union, KS M ISO14855 in Korea, and JIS K 6953 in Japan. The test-passed products are labeled as “compostable” in many countries, whereas “eco-labeled” and “biodegradable” labels are inappropriately applied in Korea and Japan (Supplementary Table S1). This can lead to overestimation of biodegradability of the labeled products.

This mini-review aims to bridge the following three gaps in biodegradable plastics by elucidating the common misconceptions and truths about biodegradation: i) the gaps among reported biodegradation level of biodegradable plastics; ii) the gaps between the biodegradation conditions in the controlled laboratory system and in the natural environment; and iii) the gaps between public perception and the actual environmental fate of biodegradable products. These gaps are critically reviewed with feasible solutions. This work will ease the assessment of biodegradable plastics and provide sound communication on corresponding claims–a prerequisite for successful market performance.

## Principle of the Biodegradation Process

The first step to bridge the gaps is to understand the biodegradation of biodegradable plastics–How then do these materials decompose in the natural environments? Biodegradation is a biological process, whereby polymers are partially or completely converted into the end products by the action of microorganisms (Song et al., 2009; Luckachan and Pillai, 2011; Soroudi and Jakubowicz, 2013). Biodegradable plastics are decomposed via four stages: biodeterioration, depolymerization, assimilation, and mineralization (Harrison et al., 2018). First, the microbial biofilms are formed on the surface of the materials, and decomposers and/or abiotic factors fragment them into tiny fractions, while polymers lose their initial physicochemical properties. Second, from the biofilm, the extracellular enzymes are excreted. Numerous enzymes specifically depolymerize polymers into smaller units such as oligomers, dimers, and monomers, reducing the molecular weight. Third, these molecules are metabolically assimilated in the cytoplasm, producing new biomass and energy as well as primary and secondary metabolites. Eventually, these metabolites are mineralized into the end products such as carbon dioxide, methane, water, and mineral salts (Lucas et al., 2008).

The biodegradation of biodegradable plastics depends on diverse factors including, but not limited to, the physicochemical structure of the materials, environmental conditions, and the microbial populations involved in the biodegradation (Massardier-Nageotte et al., 2006). In the natural ecosystem, biotic and abiotic factors synergistically decompose biodegradable plastics. Biotic factors are plastic-decomposing microorganisms such as bacteria, fungi, archaea, and algae (Lee et al., 2005; Kyrikou and Briassoulis, 2007; Gonzalez-Fernandez et al., 2015). Some examples of abiotic factors include temperature, sunlight, mechanical impact (weathering), oxygen, humidity, and acidity (Song et al., 2009; Thakur et al., 2018). Abiotic hydrolysis is the main degradation step, as humidity and temperature enable cleavage of the ester linkage (Karamanlioglu et al., 2017). The rate of hydrolysis and biodeterioration increases when the temperature exceeds the glass temperature of the polymer (Henton et al., 2005). The presence of oxygen determines the type of decomposers and biological reaction. When oxygen is available, aerobic organisms utilize the polymers as carbon and energy sources (Sudesh and Iwata, 2008). In anaerobic conditions, anaerobic organisms decompose polymers and generate biogas, mainly in the form of methane (Tokiwa et al., 2009; Mekonnen et al., 2013; Bátori et al., 2018).

It should be underlined that degradation and biodegradation are different (Shah et al., 2008; Luckachan and Pillai, 2011). Degradation of non-biodegradable plastics terminates at the fragmentation stage, generating even more persistent microplastics (Wagner et al., 2014; Auta et al., 2017; Mason et al., 2018). In contrast, biodegradation involves further biological steps, ultimately mineralizing the polymers. We suggest that the definition of biodegradable plastics be established based on their capacity to be mineralized into gaseous end products when the surrounding condition meets biodegradability requirements such as temperature, moisture, and microbial populations.

## The First gap: Differences in Reported Biodegradation of Biodegradable Plastics

**FIGURE 1:**
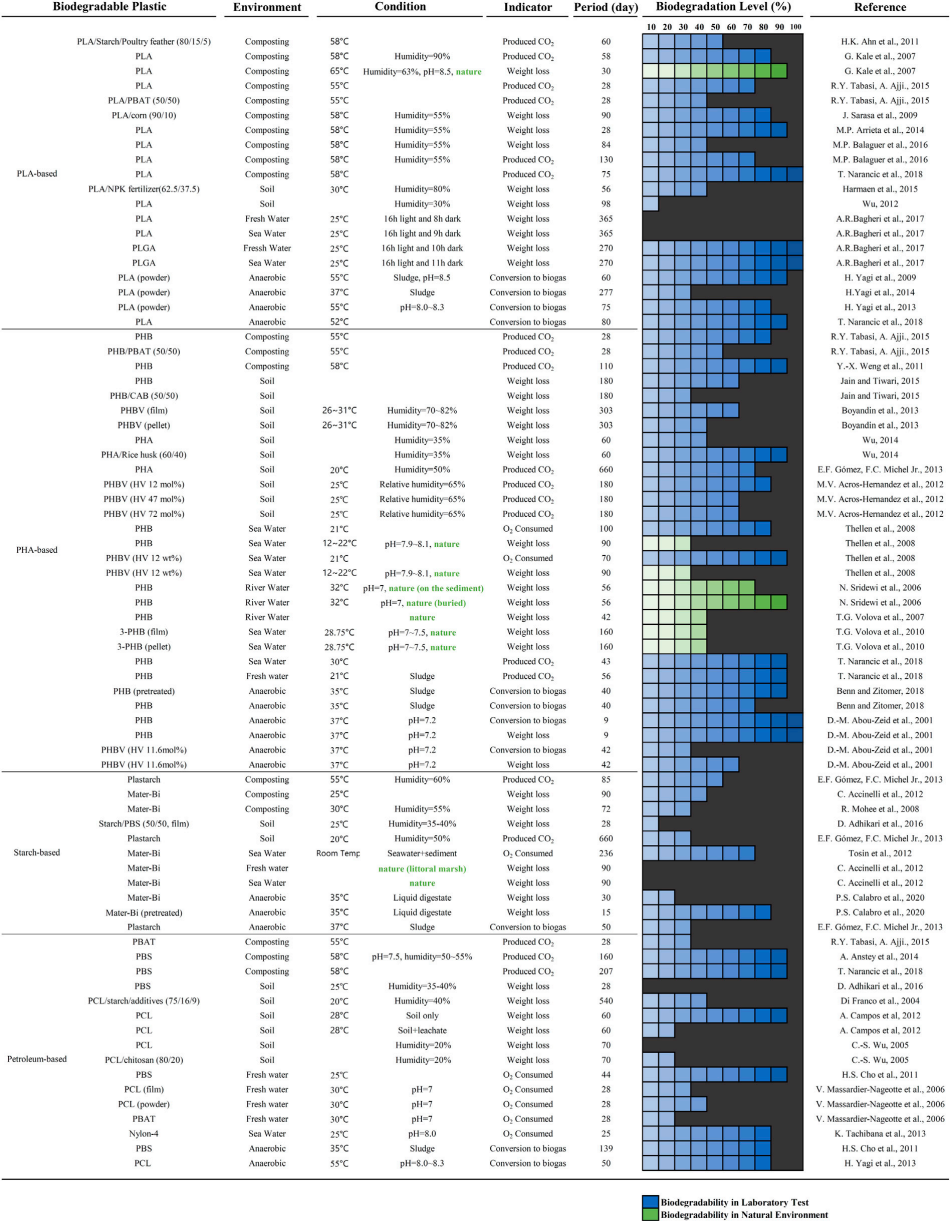
Biodegradation study results of biodegradable plastics in the laboratory (blue) and in the natural environments (green), selected by the authors. The comprehensive biodegradation data are available in the supplementary material (Supplementary Table S2). Note that the analytical analysis (averaging) was conducted based on the supplementary data set.

We collected the results of studies that quantitatively measured biodegradation level via weight loss and/or produced gaseous end products (Figure 1). Despite some variations among studies, we analyzed the biodegradation data to obtain critical insights into biodegradable plastics.

The bio-based and biodegradable poly(hydroxyalkanoates) (PHA) and PLA are most widely studied. PHA-based bioplastics are biodegradable in all indicated environments (Volova et al., 2007; Woolnough et al., 2008; Yagi et al., 2014). PLA-based bioplastics, on the other hand, are well biodegradable under industrial composting and anaerobic digesting conditions, but are hardly biodegradable in soil and aquatic environments (Pranamuda et al., 1997; Itävaara et al., 2002; Tsuji and Suzuyoshi, 2002a; Tsuji and Suzuyoshi, 2002b; Shogren et al., 2003; Martin et al., 2014). PLA requires specific high-temperature conditions and degrades through abiotic hydrolysis (Elsawy et al., 2017; Gorrasi and Pantani, 2017). On the contrary, poly(lactide-co-glycolide) (PLGA), a PLA-based copolymer, was completely biodegraded in seawater and freshwater within 270 days (Bagheri et al., 2017).

The type of environment is a significant determinant of biodegradation. Each environment has different conditions such as temperature, humidity, and microbial populations (Kale et al., 2007; Tokiwa et al., 2009). Based on comprehensive data analysis (Supplementary Table S2), we show the manifest difference in biodegradation in various environments. The average biodegradation level can be arranged in descending order as follows: industrial composting (72.3% over 75 days), anaerobic digestion (64.6% over 88 days), marine (47.1% over 155 days), soil (39.7% over 159 days), and aerobic aqueous (31.7 over 113 days) environments. Industrial composting is a highly optimized system for the biodegradation by thermophilic microorganisms (Gómez and Michel, 2013; Arrieta et al., 2014). Due to the high temperature (typically 55–60°C, Mathur, 1998) and proper water content (50–60% is appropriate for most materials, Mathur, 1998), the highest biodegradation level and shortest period are achieved under industrial composting conditions.

The microbial populations and the fraction of decomposers in the microbial community significantly differ depending on the environment. Microorganisms generally thrive where the environmental conditions suffice. Industrial composting and soil environments contain more microbes per unit than aquatic environments (Watson et al., 1977; Flemming and Wuertz, 2019; Wang et al., 2020). Even if the same material is tested under the same type of environment, the biodegradation level can vary to a great extent. For example, in one study (Boyandin et al., 2013), the weight of poly(3-hydroxybutyrate) (PHB) films buried in natural soil was reduced by 98 and 47% near Hanoi and Nha Trang, Vietnam, respectively. The higher biodegradation in the Hanoi area was attributed to richer PHA degrading microbial populations in the soil.

Although biodegradation cannot be measured via weight loss, it is widely applied in degradation tests (Shah et al., 2008). Weight loss can incorporate the influence of biodegradation, abiotic hydrolysis, and production of water-soluble products (e.g., plasticizers). In one study (Abou-Zeid et al., 2001), the weight of poly(3-hydroxybutyrate-co-3-hydroxyvalerate) (PHBV) was reduced by 60%, but only 29% of the theoretical biogas was formed. Measuring the gaseous end product by a respirometry system determines the biodegradation level precisely, although some carbons are assimilated into the new biomass (Shah et al., 2008). The biomass should be accounted for in the carbon balance during biodegradation. However, no analytical methods are available until now (Degli Innocenti and Breton, 2020). Therefore, weight loss should be applied as a biodegradation indicator only when the condition meets biodegradability requirements.

In sum, due to numerous biotic and abiotic factors being complicatedly involved in the biodegradation process, discrepancies among reported biodegradation level are inevitably present, and are difficult to standardize.

## The second gap: Disparity in Biodegradation Conditions in the Laboratory and in the Natural Environments

**FIGURE 2:**
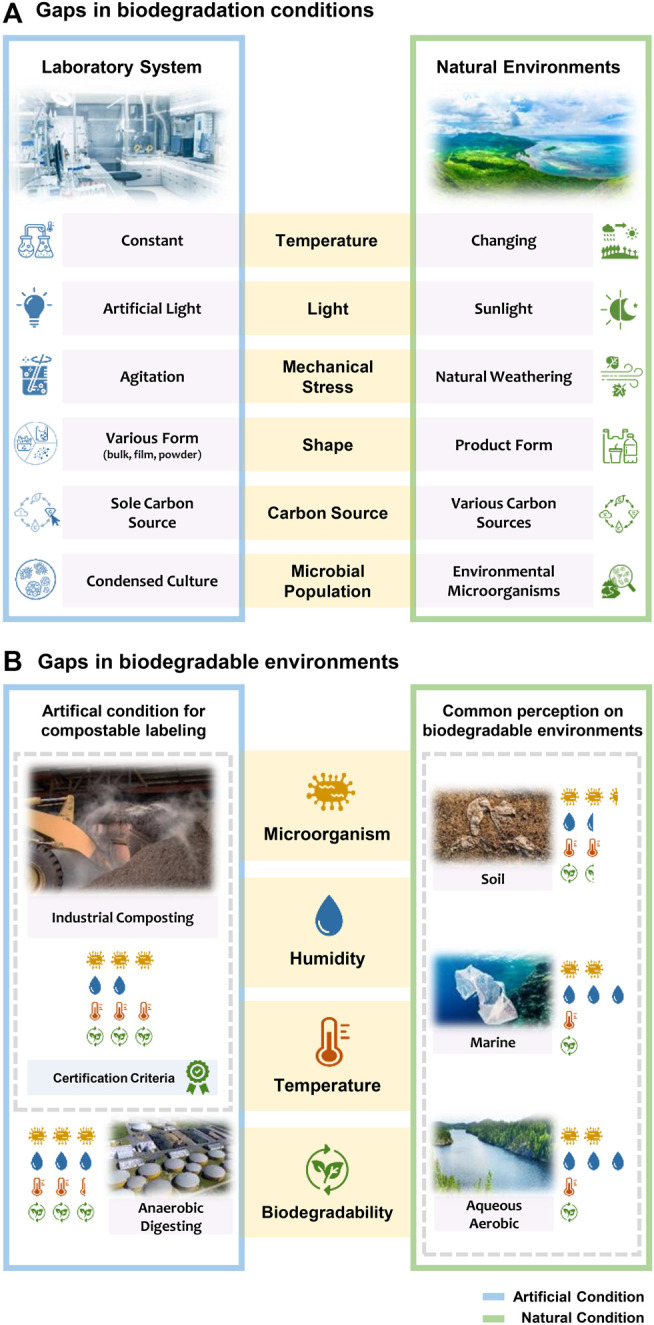
**(A)** Disparity in biodegradation conditions in the laboratory and in the natural environments. **(B)** The discrepancy between public perception and the actual biodegradability of labeled products. An industrial composting test is commonly applied for the certification, but the conditions in industrial composting facilities hardly exist in natural environments.

Biodegradability studies have been mainly conducted under laboratory systems based on test standards by simulating various biodegradation environments. However, biodegradation is highly accelerated in designed laboratory systems where the condition meets all biodegradability requirements. It is important to understand that there is a discrepancy between biodegradation conditions in the laboratory and in the natural environments (Figure 2A).

Temperature is a significant abiotic factor that influences microbial activity (Shen and Burgess, 2012; Pischedda et al., 2019), thermal degradation, and aging (Deroiné et al., 2014). Biodegradable plastics experience fluctuating temperature in nature, whereas they remain under stable temperature in a laboratory system (Rudnik and Briassoulis, 2011). For instance, in temperate regions, seasonal temperature fluctuation accelerates or delays biodegradation. Biodegradation research conducted under both conditions showed such disparity: 30°C in laboratory incubation (ASTM D6691) compared to 12–22°C in aquarium incubation subjected to continuously flowing seawater (Thellen et al., 2008).

Access to sunlight accelerates the decomposition of polymers (photodegradation). Most plastics tend to absorb high-energy radiation in the ultraviolet (UV) portion of the spectrum, which can activate their electrons to higher reactivity and cause oxidation, cleavage, and other degradation (Shah et al., 2008; Sadi et al., 2010). UV radiation can disrupt polymeric chains and embrittle the polymers by being absorbed in oxygen-containing components (Lucas et al., 2008; Rujnić-Sokele and Pilipović, 2017). Several ISO standards suggest that the samples be located under a dark or diffused light incubator. However, artificial light (i.e., a light bulb) in a laboratory system generally does not generate UV radiation. This contrasts with the conditions in a natural environment with intermittent (e.g., floating and sinking in the ocean) or long-term exposure to sunlight.

Mechanical stress is a significant abiotic parameter that affects the degradation of plastics. Weathering degradation of plastics results in their surface embrittlement and microcracking (Andrady, 2011). The free surface energy increased after weathering of PLA-lignin bioplastics (Spiridon et al., 2015). In addition, continuously pumped seawater may increase the time for microorganisms to colonize surfaces (Deroiné et al., 2014; Wright et al., 2020). Laboratory systems mimic these natural mechanical stresses by agitating incubators. However, agitation during the laboratory experiment showed little influence on biodegradation level (Briese et al., 1994).

The shape of materials considerably affects biodegradation rate (Volova et al., 2010; Boyandin et al., 2013). In the laboratory test, the samples are generally prepared in powder, film, or pellet forms for the assessment of their biodegradability. This maximizes surface area and facilitates the biodeterioration stage by providing more surface area for microorganisms to colonize, and therefore accelerates biodegradation rate (Andrady, 1994; Chinaglia et al., 2018). However, in most cases, littered products are decomposed from their original shapes, and disintegration occurs via multiple factors like weathering, UV radiation, microbial activity.

The presence of co-substrates such as food or beverage residue influences microbial activity (Andrady, 1994), as much of the plastic waste found in the environment consists of food-related packaging (Marsh and Bugusu, 2007; Heidbreder et al., 2019). These substrates shorten the lag phase of biodegradation by accelerating biofilm formation. However, in laboratory experiments, pure polymers are commonly tested. In this context, blending organic materials with biodegradable plastics influences the biodegradation rate. Biodegradation was accelerated when biodegradable plastics were blended with various materials, such as corn (Sarasa et al., 2009), poultry feather fibers (Ahn et al., 2011), rice husk (Wu, 2014), potato peel waste (Wei et al., 2015), and empty fruit bunch fiber (Wei et al., 2015).

The microbial populations differ depending on the environment. The heterogeneity of environmental microorganisms (inoculum) leads to inconsistent biodegradability test results (Haider et al., 2019). The laboratory settings are designed to highly condense or isolate decomposers since they utilize polymers as the sole carbon and energy sources (Dussud et al., 2018), thereby accelerating biodegradation. In the natural environment, however, competition within the environmental microbiome takes place due to the presence of various carbon sources and the heterogeneity of the microbial populations (Andrady, 1994).

In sum, due to the disparity of key factors, biodegradation tests under laboratory systems do not sufficiently reflect natural conditions and this can lead to overestimation of the biodegradation level of biodegradable plastics in the event of littering. Therefore, biodegradation test standards should clearly indicate the limit of representativeness and/or new specific standards should provide more options to minimize the gaps to transfer natural biodegradation to the laboratory. For instance, the temperature range should be altered depending on the geographical region. Access to sunlight and water should also be considered to imitate synergistic reactions of biotic and abiotic factors.

## The Third gap: Misconceptions and Truths on the Environmental Fate of Biodegradable Products

The general knowledge of bioplastics is low, but perception, particularly of biodegradable plastics, is positive (Lynch et al., 2017; Dilkes-Hoffman et al., 2019). Choosing biodegradable products could be adopted as a possible solution to plastic pollution rather than reducing plastic consumption (Klein et al., 2019). Marketers are keen to tout the biodegradability of materials. However, a biodegradable label on products often leads to littering wastes with the belief that biodegradable plastics will decompose naturally (Klöckner, 2013; UNEP, 2015). The truth is that they might not be as biodegradable as commonly believed (Figure 2B).

The overestimation of the biodegradability of biodegradable plastics derives from three factors: i) reported biodegradability resulted from an optimized laboratory system; ii) the overlapping term between biodegradable and compostable plastics; and iii) a lack of discrete recycling codes and treatment systems.

First, biodegradability has been tested under laboratory systems and the results are valid. However, as examined earlier, biodegradation occurs only when surrounding condition suffices biodegradability requirements. In other words, a biodegradable plastic under laboratory test may not biodegrade in natural condition. Second, many countries clearly distinguish between compostable and biodegradable products via labeling system (Supplementary Table S1). However, as compostable plastics are often coined as biodegradable polymers (Haider et al., 2019), the public easily misunderstand the biodegradability of compostable products. In addition, few are aware that compostable and biodegradable plastics are different, and the conditions in industrial composting facilities hardly exist in natural environments. Finally, recycling code (resin identification code, in some countries) on biodegradable plastics is “7”, which indicates “other polymers”. As a result, they are hardly recycled, and in turn are incinerated, landfilled or littered (Pathak et al., 2014). Incinerating is not a desirable mode for fossil-based biodegradable plastics, as it consumes a high amount of energy and emits greenhouse gases (Razza et al., 2015; Folino et al., 2020a). Also, some biodegradable products may not have completed their life cycle when landfilled (Shin et al., 1997; Quecholac-Piña et al., 2020).

PLA provides a good illustration to explain these misconceptions. PLA is derived from bio-based resources and is applied to daily commodities such as disposable packaging and cups (Karamanlioglu et al., 2017). As PLA is promoted as an eco-friendly material, the public may assume that it is naturally decomposed in the ocean. However, PLA is well biodegradable only under industrial composting and anaerobic digesting conditions (Supplementary Table S2). Even if products are labeled compostable (as in most Western countries), many people might be confused between compostable and biodegradable plastics. Furthermore, the recycling code on PLA products is generally “7”, which may lead most littered PLA products to not being properly treated.

Therefore, we suggest that a separate recycling code for compostable plastics be established to ensure that these plastics end up their life cycle in an industrial composting facility, and littering should be the last resort. Furthermore, following the growing production trend of biodegradable plastics, separate collection systems and treatment facilities should be built, making so-called biodegradable products genuinely biodegradable. Education on biodegradable plastics should be provided so that the public can make informed decisions (UNEP, 2015). For instance, it should be understood that industrial composting test does not necessarily guarantee biodegradation in natural conditions, especially in aquatic environments. The labeling on compostable products should clearly indicate that they are only biodegradable in an industrial composting facility. Society should have access to reliable, authoritative, and clear guidance on what terms such as “compostable” or “biodegradable” actually mean. The national legalization on the i) definition, ii) classification, iii) labeling, iv) collection system, and v) treatment guideline will enhance public awareness of biodegradable plastics and eliminate misconceptions.

## Discussion and Outlook

Biodegradable polymers are only beneficial when they can actually biodegrade (Gross and Kalra, 2002). Bridging the aforementioned three gaps will enhance sound communication on biodegradable plastics, eliminating confusion and misconceptions. Understanding the truths about biodegradable plastics will provide support for the progressive substitution of conventional plastics with biodegradable plastics. There are also other anticipated outcomes: i) Products with high biodegradability will be promoted in the market. ii) Policies on eco-friendlier and sound design will be established as well as financial incentives. iii) The development of biodegradation accelerating technology will be triggered. iv) Waste littering based on the false belief in the biodegradability of biodegradable plastics will be minimized. v) Bridging the gaps in biodegradable plastics will open up a sustainable future.
