# Supplementary material for: Bridging Three Gaps in Biodegradable Plastics: Misconceptions and Truths About Biodegradation
Source: Front Chem. 2021 May 14;9:671750. doi: 10.3389/fchem.2021.671750 (PMC8160376; doi:10.3389/fchem.2021.671750)
Supplement: Supplementary file 1 [file DataSheet1.docx]

Supplementary Material

# Supplementary Data

Supplementary Material should be uploaded separately on submission. Please include any supplementary data, figures and/or tables. All supplementary files are deposited to FigShare for permanent storage and receive a DOI.

Supplementary material is not typeset so please ensure that all information is clearly presented, the appropriate caption is included in the file and not in the manuscript, and that the style conforms to the rest of the article. To avoid discrepancies between the published article and the supplementary material, please do not add the title, author list, affiliations or correspondence in the supplementary files.

# Supplementary Figures and Tables

**2.1 Supplementary Table S1.** Biodegradation Certification Systems

1. Certifications based on industrial composting test

| **Country** | **Certification** | | **Label** |
| --- | --- | --- | --- |
| European  Union | **Std.**  **Name**  **Assoc.** | DIN EN 13432  OK Compost Industrial / Seedling  TÜV AUSTRIA, DIN CERTCO | 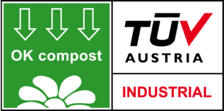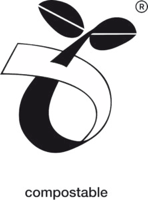 |
|  | **Std.**  **Name**  **Assoc.** | AS 5810(2010), NF T 51800(2015), prEN 17427(2020)  OK Compost Home / DIN-Geprüft Home Compostable  TÜV AUSTRIA / DIN CERTCO | 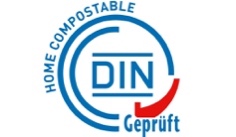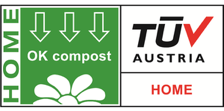 |
| Germany | **Std.**  **Name**  **Assoc.** | DIN EN 13432  DIN-Geprüft  DIN CERTCO | 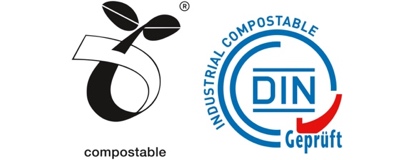 |
| Italy | **Std.**  **Name**  **Assoc.** | EN 13432  Compostable CIC  The Italian Composting and Biogas Association | 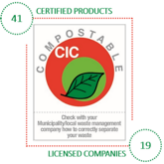 |
| United States | **Std.**  **Name**  **Assoc.** | ASTM 5338, ASTM 6400, ASTM 6868  BPI Certification Mark  Biodegradable Products Institute | 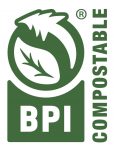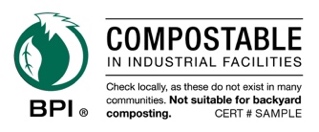 |
| Australia | **Std.**  **Name**  **Assoc.** | AS 4736  Seedling  Australasian Bioplastics Association, DIN CERTCO | 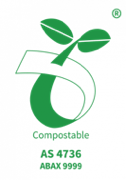 |
|  | **Std.**  **Name**  **Assoc.** | AS5810(2010)  Home Compostable  Australasian Bioplastics Association, DIN CERTCO | 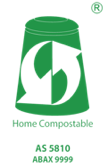 |
| Korea | **Std.**  **Name**  **Assoc.** | KS M ISO 14855  Korea Eco-Label  Korea Environmental Industry & Technology Institute | 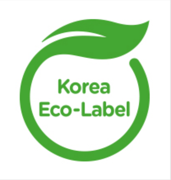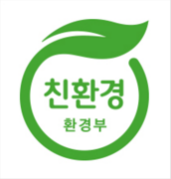 |
| Japan | **Std.**  **Name**  **Assoc.** | JIS K 6950, JIS K 6951, JIS K 6953-1, JIS K 6955  GreenPla  Japan Bioplastics Association (JPBA) | 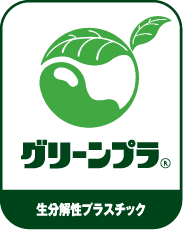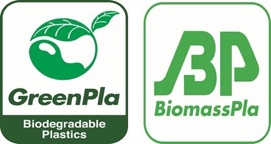 |
| China | **Std.**  **Name**  **Assoc.** | GB/T 19276.1, GB/T 19276.2, GB/T 32106 GB/T 19277.1(2011)  Biodegradable Plastic Logo  China National Light Industry Council | 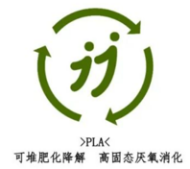 |

2. Certifications based on soil, marine, aquatic test

| **Country** | **Certification** | | **Label** |
| --- | --- | --- | --- |
| Belgium | **Std.**  **Name**  **Assoc.** | TÜV AUSTRIA’s Own Standard  OK Biodegradable Water, OK Biodegradable Soil,  OK Biodegradable Marine  TÜV AUSTRIA | 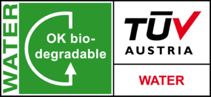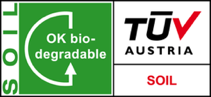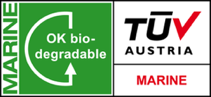 |
| Germany | **Std.**  **Name**  **Assoc.** | DIN EN 17033  DIN-Geprüft biodegradable in soil  DIN CERTCO | 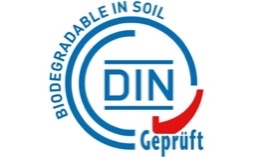 |

**Supplementary Table S2.** Biodegradation Study Results

1. PLA-based bioplastics

| **Bioplastic** | **Environment** | **Condition** | | **Indicator** | **Period**  **(day)** | **Biodegradability (%)** | **Reference** |
| --- | --- | --- | --- | --- | --- | --- | --- |
|  |  | **Temp. (℃)** | **etc** |  |  |  |  |
| PLA | Composting | 58 |  | CO_2_ produced | 60 | 13 | H.K. Ahn et al., 2011 |
| PLA/Starch/Poultry feather (80/15/5) | Composting | 58 |  | CO_2_ produced | 60 | 53 | H.K. Ahn et al., 2011 |
| PLA | Composting | 58 | Humidity=90% | CO_2_ produced | 58 | 84 | G. Kale et al., 2007 |
| PLA | Composting | 65 | **Nature**,  pH=8.5, humidity=63% | Weight loss | 30 | >90 | G. Kale et al., 2007 |
| PLA | Composting | 55 |  | CO_2_ produced | 28 | 70 | R.Y. Tabasi, A. Ajji., 2015 |
| PLA/PBAT (50/50) | Composting | 55 |  | CO_2_ produced | 28 | 43~45 | R.Y. Tabasi, A. Ajji., 2015 |
| PLA | Composting | 55 |  | Weight loss | 30 | 60 | M. Mihai et al., 2014 |
| PLA/Wood fiber (70/30) | Composting | 55 |  | Weight loss | 30 | 40 | M. Mihai et al., 2014 |
| PLA (with foaming agent; PS) | Composting | 58 | Humidity=55% | Weight loss | 90 | 63.6 | J. Sarasa et al., 2009 |
| PLA/corn (90/10) | Composting | 58 | Humidity=55% | Weight loss | 90 | 79.7 | J. Sarasa et al., 2009 |
| PLA | Composting | 58 | Humidity=55% | Weight loss | 28 | >90 | M.P. Arrieta et al., 2014 |
| plasticized PLA (with ATBC) | Composting | 58 | Humidity=55% | Weight loss | 28 | >90 | M.P. Arrieta et al., 2014 |
| plasticized PLA (with PEG) | Composting | 58 | Humidity=55% | Weight loss | 28 | >90 | M.P. Arrieta et al., 2014 |
| plasticized PLA/PHB  (75/25, with ATBC) | Composting | 58 | Humidity=55% | Weight loss | 28 | >90 | M.P. Arrieta et al., 2014 |
| plasticized PLA/PHB  (75/25, with PEG) | Composting | 58 | Humidity=55% | Weight loss | 35 | >90 | M.P. Arrieta et al., 2014 |
| PLA | Composting | 58 | Humidity=55% | Weight loss | 84 | 42.3 | M.P. Balaguer et al., 2016 |
| PLA | Composting | 58 | Humidity=55% | CO_2_ produced | 130 | >70 | M.P. Balaguer et al., 2016 |
| PLA + Clay1 | Composting | 58 | Humidity=55% | Weight loss | 84 | 41.6 | M.P. Balaguer et al., 2016 |
| PLA + Clay1 | Composting | 58 | Humidity=55% | CO_2_ produced | 130 | >90 | M.P. Balaguer et al., 2016 |
| PLA + Nano-CaCO₃ | Composting | 58 | Humidity=55% | Weight loss | 84 | 40.8 | M.P. Balaguer et al., 2016 |
| PLA + Nano-CaCO₃ | Composting | 58 | Humidity=55% | CO_2_ produced | 130 | >90 | M.P. Balaguer et al., 2016 |
| PLA + Nano-SiO₂ | Composting | 58 | Humidity=55% | Weight loss | 84 | 43.8 | M.P. Balaguer et al., 2016 |
| PLA + Nano-SiO₂ | Composting | 58 | Humidity=55% | CO_2_ produced | 130 | >80 | M.P. Balaguer et al., 2016 |
| PLA | Composting | 58 |  | CO_2_ produced | 75 | 100 | T. Narancic et al., 2018 |
| PLA/PCL (80/20) | Composting | 58 |  | CO_2_ produced | 75 | 100 | T. Narancic et al., 2018 |
| PLA/PBS (80/20) | Composting | 58 |  | CO_2_ produced | 75 | 100 | T. Narancic et al., 2018 |
| PLA/PHB (80/20) | Composting | 58 |  | CO_2_ produced | 75 | 98 | T. Narancic et al., 2018 |
| PLA | Soil |  | Humidity=30% | Weight loss | 98 | 12 | Wu, 2012 |
| PLA/Sisal (80/20) | Soil |  | Humidity=30% | Weight loss | 98 | 47~49 | Wu, 2012 |
| PLA/Sisal (60/40) | Soil |  | Humidity=30% | Weight loss | 98 | 67~70 | Wu, 2012 |
| PLA/NPK fertilizer (62.5/37.5) | Soil | 30 | Humidity=80% | Weight loss | 56 | 35-40 | Harmaen et al., 2015 |
| PLA/NPK fertilizer/EFB fiber (37.5/37.5/30) | Soil | 30 | Humidity=80% | Weight loss | 56 | 45 | Harmaen et al., 2015 |
| PLA | Soil | 25 | Humidity=35~40% | Decrease in soil TC | 28 | 13.8 | D. Adhikari et al., 2016 |
| PLA | Fresh Water | 30 | pH=7, sludge | O_2_ consumed | 28 | 3.7 | V. Massardier-Nageotte et al., 2006 |
| PLA | Fresh Water | 25 | 16h light and 8h dark | Weight loss | 365 | <2 | A.R.Bagheri et al., 2017 |
| PLA | Sea Water | 25 | 16h light and 8h dark | Weight loss | 365 | <2 | A.R.Bagheri et al., 2017 |
| PLGA | Fresh Water | 25 | 16h light and 8h dark | Weight loss | 270 | 100 | A.R.Bagheri et al., 2017 |
| PLGA | Sea Water | 25 | 16h light and 8h dark | Weight loss | 270 | 100 | A.R.Bagheri et al., 2017 |
| PLA | Anaerobic | 55 | pH=8.5, sludge | Conversion to biogas | 60 | 90 | H. Yagi et al., 2009 |
| PLA | Anaerobic | 37 | Sludge | Conversion to biogas | 277 | 49 | H. Yagi et al., 2009 |
| PLA | Anaerobic | 37 | Sludge | Conversion to biogas | 277 | 29 | H. Yagi et al., 2009 |
| PLA | Anaerobic | 55 | pH=8.0~8.3 | Conversion to biogas | 75 | 75 | H. Yagi et al., 2009 |
| PLA | Anaerobic | 55 | pH=8.0 | Conversion to biogas | 80 | 83 | H. Yagi et al., 2009 |
| PLA | Anaerobic | 52 |  | Conversion to biogas | 80 | 88 | T. Narancic et al., 2018 |
| PLA/PCL (80/20) | Anaerobic | 52 |  | Conversion to biogas | 121 | 90 | T. Narancic et al., 2018 |
| PLA/PBS (80/20) | Anaerobic | 52 |  | Conversion to biogas | 121 | 85 | T. Narancic et al., 2018 |
| PLA/PHB (80/20) | Anaerobic | 52 |  | Conversion to biogas | 80 | 100 | T. Narancic et al., 2018 |

2. PHA-based bioplastics

| **Bioplastic** | **Environment** | **Condition** | | **Indicator** | **Period**  **(day)** | **Biodegradability (%)** | **Reference** |
| --- | --- | --- | --- | --- | --- | --- | --- |
|  |  | **Temp.(℃)** | **Etc** |  |  |  |  |
| PHB | Composting | 55 |  | CO_2_ produced | 28 | 78~80 | R.Y. Tabasi, A. Ajji., 2015 |
| PHB/PBAT (50/50) | Composting | 55 |  | CO_2_ produced | 28 | 47~48 | R.Y. Tabasi, A. Ajji., 2015 |
| PHB | Composting | 58 |  | CO_2_ produced | 110 | 90 | Y.-X. Weng et al., 2011 |
| PHB | Composting | 58 |  | CO_2_ produced | 45 | 100 | T. Narancic et al., 2018 |
| PHB/PCL (60/40) | Composting | 58 |  | CO_2_ produced | 46 | 100 | T. Narancic et al., 2018 |
| PHB/PBS (50/50) | Composting | 58 |  | CO_2_ produced | 88 | 100 | T. Narancic et al., 2018 |
| PHB | Soil |  |  | Weight loss | 180 | 64.3 | Jain and Tiwari, 2015 |
| PHB/CAB (50/50) | Soil |  |  | Weight loss | 180 | 31.5 | Jain and Tiwari, 2015 |
| PHB (film) | Soil | 26~31 | Humidity=70~82% | Weight loss | 303 | 98 | Boyandin et al., 2013 |
| PHB (pellet) | Soil | 26~31 | Humidity=70~82% | Weight loss | 303 | 55 | Boyandin et al., 2013 |
| PHBV (film) | Soil | 26~31 | Humidity=70~82% | Weight loss | 303 | 61 | Boyandin et al., 2013 |
| PHBV (pellet) | Soil | 26~31 | Humidity=70~82% | Weight loss | 303 | 35 | Boyandin et al., 2013 |
| PHB (film) | Soil | 27~30 | Humidity=78~84% | Weight loss | 385 | 47 | Boyandin et al., 2013 |
| PHB (pellet) | Soil | 27~30 | Humidity=78~84% | Weight loss | 385 | 28 | Boyandin et al., 2013 |
| PHBV (film) | Soil | 27~30 | Humidity=78~84% | Weight loss | 385 | 14 | Boyandin et al., 2013 |
| PHBV (pellet) | Soil | 27~30 | Humidity=78~84% | Weight loss | 385 | 8 | Boyandin et al., 2013 |
| PHA | Soil |  | Humidity=35% | Weight loss | 60 | 35 | Wu, 2014 |
| PHA/Rice husk (60/40) | Soil |  | Humidity=35% | Weight loss | 60 | 90 | Wu, 2014 |
| PHA | Soil | 20 | Humidity=50% | CO_2_ produced | 660 | 69.2 | E.F. Gomez, F.C. Michel Jr., 2013 |
| PHB | Soil | 25 | pH=6.5,  water content=16% | Weight loss | 9 | 40 | CA Woolnough et al., 2008 |
| P(HB-co-8HV) | Soil | 25 | pH=6.5,  water content=16% | Weight loss | 9 | 50 | CA Woolnough et al., 2008 |
| PHB | Soil | 25 | pH=6.5,  water content=16% | Weight loss | 9 | 18 | CA Woolnough et al., 2008 |
| P(HB-co-8HV) | Soil | 25 | pH=6.5,  water content=16% | Weight loss | 9 | 10 | CA Woolnough et al., 2008 |
| PHBV (HV 12 mol%) | Soil | 25 | Relative humidity = 65% | CO_2_ produced | 180 | 75 | M.V. Acros-Hernandez et al., 2012 |
| PHBV (HV 43 mol%) | Soil | 25 | Relative humidity = 65% | CO_2_ produced | 180 | 70 | M.V. Acros-Hernandez et al., 2012 |
| PHBV (HV 47 mol%) | Soil | 25 | Relative humidity = 65% | CO_2_ produced | 180 | 59 | M.V. Acros-Hernandez et al., 2012 |
| PHBV (HV 52 mol%) | Soil | 25 | Relative humidity = 65% | CO_2_ produced | 180 | 74 | M.V. Acros-Hernandez et al., 2012 |
| PHBV (HV 64 mol%) | Soil | 25 | Relative humidity = 65% | CO_2_ produced | 180 | 60 | M.V. Acros-Hernandez et al., 2012 |
| PHBV (HV 72 mol%) | Soil | 25 | Relative humidity = 65% | CO_2_ produced | 180 | 62 | M.V. Acros-Hernandez et al., 2012 |
| PHB | Soil | 25 |  | CO_2_ produced | 136 | 100 | T. Narancic et al., 2018 |
| PHB/PCL (60/40) | Soil | 25 |  | CO_2_ produced | 256 | 100 | T. Narancic et al., 2018 |
| PHB | Sea Water | 21 |  | O_2_ consumed | 100 | 80 | Thellen et al., 2008 |
| PHB | Sea Water | 12~22 | **Nature**, pH=7.9~8.1 | Weight loss | 90 | 30 | Thellen et al., 2008 |
| PHBV (HV 12wt%) | Sea Water | 21 |  | O_2_ consumed | 70 | 90 | Thellen et al., 2008 |
| PHBV (HV 12wt%) | Sea Water | 12~22 | **Nature**, pH=7.9~8.1 | Weight loss | 90 | 33 | Thellen et al., 2008 |
| PHB | River Water | 32 | pH=7,  **Nature (on the sediment)** | Weight loss | 56 | 70 | N. Sridewi et al., 2006 |
| PHB | River Water | 32 | pH=7,  **Nature (buried)** | Weight loss | 56 | >90 | N. Sridewi et al., 2006 |
| PHB | Fresh water | 25 | 16h light and 8h dark | Weight loss | 365 | 8.5 | A.R.Bagheri et al., 2017 |
| PHB | Sea Water | 25 | 16h light and 8h dark | Weight loss | 365 | 8.5 | A.R.Bagheri et al., 2017 |
| PHB | River Water |  | **Nature**, in 1999 | Weight loss | 42 | 43.5 | T.G. Volova et al., 2007 |
| PHB | River Water |  | **Nature**, in 2000 | Weight loss | 31 | 34.6 | T.G. Volova et al., 2007 |
| 3-PHB (film) | Sea Water | 28.75 | **Nature**, pH=7~7.5 | Weight loss | 160 | 42 | T.G. Volova et al., 2010 |
| 3-PHB/PHV (film) | Sea Water | 28.75 | **Nature**, pH=7~7.5 | Weight loss | 160 | 46 | T.G. Volova et al., 2010 |
| 3-PHB (pellet) | Sea Water | 28.75 | **Nature**, pH=7~7.5 | Weight loss | 160 | 38 | T.G. Volova et al., 2010 |
| 3-PHB/PHV (pellet) | Sea Water | 28.75 | **Nature**, pH=7~7.5 | Weight loss | 160 | 13 | T.G. Volova et al., 2010 |
| PHB | Fresh water | 21 |  | CO_2_ produced | 56 | 90 | T. Narancic et al., 2018 |
| PHB | Sea Water | 30 |  | CO_2_ produced | 43 | 90 | T. Narancic et al., 2018 |
| PHB/PCL (60/40) | Sea Water | 30 |  | CO_2_ produced | 56 | 86 | T. Narancic et al., 2018 |
| PHB | Anaerobic | 37 | Sludge | Conversion to biogas | 26 | 93 | H.Yagi et al., 2014 |
| PHB | Anaerobic | 37 | Sludge | Conversion to biogas | 26 | 92 | H.Yagi et al., 2014 |
| PHB (pretreated) | Anaerobic | 35 | Sludge | Conversion to biogas | 40 | 91 | Benn and Zitomer, 2018 |
| PHB | Anaerobic | 35 | Sludge | Conversion to biogas | 40 | 67 | Benn and Zitomer, 2018 |
| PHB | Anaerobic | 55 | pH=8.0~8.3 | Conversion to biogas | 14 | 90 | H. Yagi et al., 2013 |
| PHB | Anaerobic | 37 | pH=7.2 | Conversion to biogas | 9 | 100 | D.-M. Abou-Zeid et al., (2001) |
| PHB | Anaerobic | 37 | pH=7.2 | Weight loss | 9 | 100 | D.-M. Abou-Zeid et al., (2001) |
| PHBV (11.6mol%) | Anaerobic | 37 | pH=7.2 | Conversion to biogas | 42 | 30 | D.-M. Abou-Zeid et al., (2001) |
| PHBV (11.6mol%) | Anaerobic | 37 | pH=7.2 | Weight loss | 42 | 60 | D.-M. Abou-Zeid et al., (2001) |
| PHB | Anaerobic | 52 |  | Conversion to biogas | 127 | 92 | T. Narancic et al., 2018 |
| PHB/PCL (60/40) | Anaerobic | 52 |  | Conversion to biogas | 80 | 100 | T. Narancic et al., 2018 |
| PHB/PBS (50/50) | Anaerobic | 52 |  | Conversion to biogas | 121 | 80 | T. Narancic et al., 2018 |

3. Starch-based bioplastics

| **Bioplastic** | **Environment** | **Condition** | | **Indicator** | **Period**  **(day)** | **Biodegradability (%)** | **Reference** |
| --- | --- | --- | --- | --- | --- | --- | --- |
|  |  | **Temp.(℃)** | **etc** |  |  |  |  |
| Plastarch | Composting | 55 | Humidity=60% | CO_2_ produced | 85 | 50 | E.F. Gómez, F.C. Michel Jr., 2013 |
| Mater-Bi | Composting | 25 |  | Weight loss | 90 | 43 | C. Accinelli et al., 2012 |
| Mater-Bi | Composting | 30 | Humidity=55% | Weight loss | 72 | 26.9 | R. Mohee et al., 2008 |
| Cassava starch/glycerol (3/1) | Composting | Room Temp. | *Aspergillus niger* isolate | Weight loss | 10 | 29.89 | R.C. Nissa et al., 2018 |
| Plastarch | Soil | 20 | Humidity=50% | CO_2_ produced | 660 | 31.3 | E.F. Gomez, F.C. Michel Jr. |
| Mater-Bi | Soil | 25 |  | Weight loss | 90 | 37 | C. Accinelli et al., 2012 |
| Mater-Bi | Soil |  | **Nature** | Weight loss | 90 | 3.4 | C. Accinelli et al., 2012 |
| Starch/PBS (50/50, film) | Soil | 25 | Humidity=35~40% | Weight loss | 28 | 7.2 | D. Adhikari et al., 2016 |
| Starch/PBS (50/50, powder) | Soil | 25 | Humidity=35~40% | Decrease in soil TC | 28 | 24.4 | D. Adhikari et al., 2016 |
| Cassava starch/glycerol (3/1) | Soil | Room Temp. | *Aspergillus niger* isolate | Weight loss | 10 | 29.89 | R.C. Nissa et al., 2018 |
| Mater-bi | Fresh water | 30 | pH=7 | O_2_ consumed | 28 | 42.8 | V. Massardier-Nageotte et al., 2006 |
| Mater-bi | Sea water | Room Temp. | Seawater+sediments | O_2_ consumed | 236 | 68.9 | Tosin et al., 2012 |
| Mater-bi | Fresh water | 25 |  | Weight loss | 90 | 1.6 | C. Accinelli et al., 2012 |
| Mater-bi | Fresh water |  | **Nature (littoral marsh)** | Weight loss | 90 | 1.5 | C. Accinelli et al., 2012 |
| Mater-bi | Sea water | 25 |  | Weight loss | 90 | 1.7 | C. Accinelli et al., 2012 |
| Mater-bi | Sea water |  | **Nature** | Weight loss | 90 | 4.5 | C. Accinelli et al., 2012 |
| Cassava starch/glycerol (3/1) | Fresh water | 30 | *Aspergillus niger* isolate | Weight loss | 10 | 20.18 | R.C. Nissa et al., 2018 |
| Cassava starch/glycerol (3/1) | Fresh water | 30 | *Aspergillus niger* isolate | Weight loss | 10 | 11.46 | R.C. Nissa et al., 2018 |
| Mater-bi | Anaerobic | 35 | pH=7, sludge | Conversion to biogas | 28 | 23 | Massardier-Nageotte et al., 2006 |
| Mater-bi | Anaerobic | 35 | Liquid digestate | Weight loss | 30 | 24.1 | P.S. Calabro et al., 2020 |
| Mater-bi | Anaerobic | 55 | Liquid digestate | Weight loss | 30 | 37 | P.S. Calabro et al., 2020 |
| Mater-bi (pretreated) | Anaerobic | 35 | Liquid digestate, | Weight loss | 15 | 78.2 | P.S. Calabro et al., 2020 |
| Plastarch | Anaerobic | 37 | Sludge | Conversion to biogas | 50 | 26.4 | Gómez and Michel, 2013 |

4. Petroleum-based bioplastics

| **Bioplastic** | **Environment** | **Condition** | | **Indicator** | **Period**  **(day)** | **Biodegradability (%)** | **Reference** |
| --- | --- | --- | --- | --- | --- | --- | --- |
|  |  | **Temp.(℃)** | **Etc** |  |  |  |  |
| PBAT | Composting | 55 |  | CO_2_ produced | 28 | 34~35 | R.Y. Tabasi, A. Ajji., 2015 |
| PBS | Composting | 58 | pH=7.5, humidity=50~55% | CO_2_ produced | 160 | 90 | A. Anstey et al., 2014 |
| PBS/soy meal (75/25) | Composting | 58 | pH=7.5, humidity=50~55% | CO_2_ produced | 140 | 90 | A. Anstey et al., 2014 |
| PBS/canola meal (75/25) | Composting | 58 | pH=7.5, humidity=50~55% | CO_2_ produced | 160 | 90 | A. Anstey et al., 2014 |
| PBS/corn gluten meal (75/25) | Composting | 58 | pH=7.5, humidity=50~55% | CO_2_ produced | 160 | 90 | A. Anstey et al., 2014 |
| PBS/switch grass (75/25) | Composting | 58 | pH=7.5, humidity=50~55% | CO_2_ produced | 170 | 90 | A. Anstey et al., 2014 |
| PCL | Composting | 50 | pH=7~8.5 | CO_2_ produced | 6 | 38 | K. Nakasaki et al., 2006 |
| PCL | Composting | 58 |  | CO_2_ produced | 45 | 100 | T. Narancic et al., 2018 |
| PBS | Composting | 58 |  | CO_2_ produced | 207 | 92 | T. Narancic et al., 2018 |
| PCL/starch/additives (75/16/9) | Soil | 20 | Humidity=40% | Weight loss | 540 | 39 | Di Franco et al., 2004 |
| PBS (film) | Soil | 25 | Humidity=35~40% | Weight loss | 28 | 1.2 | D. Adhikari et al., 2016 |
| PBS (powder) | Soil | 25 | Humidity=35~40% | Decrease in soil TC | 28 | 16.8 | D. Adhikari et al., 2016 |
| PVC | Soil | 28 | Soil only | Weight loss | 120 | 1 | A. Campos et al, 2012 |
| PVC | Soil | 28 | Soil + leachate | Weight loss | 120 | 1.3 | A. Campos et al, 2012 |
| PCL | Soil | 28 | Soil only | Weight loss | 60 | 89.7 | A. Campos et al, 2012 |
| PCL | Soil | 28 | Soil + leachate | Weight loss | 60 | 22 | A. Campos et al, 2012 |
| PVC/PCL (1/1) | Soil | 28 | Soil only | Weight loss | 120 | 1.9 | A. Campos et al, 2012 |
| PVC/PCL (1/1) | Soil | 28 | Soil + leachate | Weight loss | 120 | 1.6 | A. Campos et al, 2012 |
| PCL | Soil |  | Humidity=20% | Weight loss | 70 | <5 | C.-S. Wu, 2005 |
| PCL/chitosan (90/10) | Soil |  | Humidity=20% | Weight loss | 70 | 10 | C.-S. Wu, 2005 |
| PCL/chitosan (80/20) | Soil |  | Humidity=20% | Weight loss | 70 | 20 | C.-S. Wu, 2005 |
| PCL-g-AA | Soil |  | Humidity=20% | Weight loss | 70 | <5 | C.-S. Wu, 2005 |
| PCL-g-AA/chitosan (90/10) | Soil |  | Humidity=20% | Weight loss | 70 | <10 | C.-S. Wu, 2005 |
| PCL-g-AA/chitosan (80/20) | Soil |  | Humidity=20% | Weight loss | 70 | 15 | C.-S. Wu, 2005 |
| Nylon-4 | Soil | 25 | pH=7.5~7.6, humidity = 80% | Weight loss | 120 | 100 | K. Hashimoto et al, 2002 |
| PCL | Soil | 25 |  | CO_2_ produced | 136 | 92 | T. Narancic et al., 2018 |
| PCL | Fresh Water | 30 | pH=7, sludge | O_2_ consumed | 28 | 34.8 | V. Massardier-Nageotte et al., 2006 |
| PCL | Fresh Water | 30 | pH=7, sludge | O_2_ consumed | 28 | 37.7 | V. Massardier-Nageotte et al., 2006 |
| PBAT | Fresh Water | 30 | pH=7, sludge | O_2_ consumed | 28 | 15.1 | V. Massardier-Nageotte et al., 2006 |
| PBS | Fresh Water | 25 | Sludge | O_2_ consumed | 44 | 88 | H.S. Cho et al., 2011 |
| PCL/starch/aliphatic polyesters (55/30/15) | Fresh Water | 25 | Sludge | O_2_ consumed | 80 | 31 | H.S. Cho et al., 2011 |
| Nylon-4 | Sea Water | 25 | pH=8 | O_2_ consumed | 25 | 80 | K. Tachibana et al., 2013 |
| Nylon-4 | Fresh Water | 25 | pH=6.9, sludge | CO_2_ produced | 28 | 49 | K. Hashimoto et al., 2002 |
| PCL | Fresh Water | 25 | 16h light and 8h dark | Weight loss | 365 | <2 | A.R.Bagheri et al., 2017 |
| PCL | Sea Water | 25 | 16h light and 8h dark | Weight loss | 365 | <2 | A.R.Bagheri et al., 2017 |
| PCL | Sea Water | 30 |  | CO_2_ produced | 56 | 80 | T. Narancic et al., 2018 |
| PCL | Anaerobic | 37 | Sludge | Conversion to biogas | 277 | 22 | H.Yagi et al., 2014 |
| PCL | Anaerobic | 37 | Sludge | Conversion to biogas | 277 | 3 | H.Yagi et al., 2014 |
| PBS | Anaerobic | 35 | Sludge | Conversion to biogas | 139 | 83 | H.S. Cho et al., 2011 |
| PCL/starch (55/35) | Anaerobic | 35 | Sludge | Conversion to biogas | 100 | 2 | H.S. Cho et al., 2011 |
| PCL | Anaerobic | 55 | pH=8.0~8.3 | Conversion to biogas | 50 | 80 | H. Yagi et al., 2013 |
| PCL | Anaerobic | 37 | pH=7.2 | Conversion to biogas | 42 | 30 | D.-M. Abou-Zeid et al., 2001 |
| PCL | Anaerobic | 37 | pH=7.2 | Weight loss | 70 | 8 | D.-M. Abou-Zeid et al., 2001 |
| PCL | Anaerobic | 52 |  | Conversion to biogas | 127 | 95 | T. Narancic et al., 2018 |
